# Risk Assessment Arising from the Exposure of Terrestrial Vertebrates to Soil Contamination: Learning from Field Lizards of the *Podarcis* Genus

**DOI:** 10.3390/jox15010021

**Published:** 2025-02-01

**Authors:** Rosaria Scudiero, Teresa Chianese, Patrizia Cretì, Luigi Rosati

**Affiliations:** 1 Department of Biology, University of Naples Federico II, Via Cinthia, 80126 Napoli, Italy; teresa.chianese2@unina.it (T.C.); luigi.rosati@unina.it (L.R.); 2 Department of Biological and Environmental Sciences and Technologies, University of Salento, Via Monteroni, 73100 Lecce, Italy; patrizia.creti@unisalento.it

**Keywords:** biomonitoring, cadmium, endocrine disrupting chemicals, pesticides, *Podarcis*, reproductive toxicology, soil pollution

## Abstract

The soil environment has been considered capable of storing toxic substances without serious consequences for the inhabitants since plants are able to bioaccumulate pollutants without compromising their survival. The application of chemicals to increase soil productivity and the dumping of waste have worsened soil quality. Recently, following a greater awareness of the importance of monitoring the damage deriving from the consumption of contaminated crops for humans and of the protection of biodiversity, studies aimed at identifying the effects of soil contamination on terrestrial animals have increased considerably. Studies using field lizards as model organisms fit into this scenario; this research has shed light on the uptake, accumulation, and toxicity of soil pollutants on reptiles. This review summarizes data collected on lizards of the *Podarcis* genus, a group of resilient wild species capable of living in both pristine and anthropized areas; the data reveal that many of the effects recorded in lizard tissues at the molecular, biochemical, and histological levels are independent of the chemical composition of the contaminants and are mostly linked to the type of cellular response. Overall, these studies confirm *Podarcis* lizards as a good model system in ecotoxicological and cytotoxicological research, providing an accurate description of the effects of pollutants, clarifying the defense mechanisms activated in relation to different exposure routes and, finally, providing predictive information on the risks faced by other animals. Since the effects recorded in lizards have often also been observed in mammals, it can be concluded that the results obtained from studies on these animals can be translated to other terrestrial vertebrates, including mammals.

## 1. Introduction

Potentially harmful elements are continuously released into the environment. Although all environmental compartments (air, water, and soil) can be contaminated, for many years, greater attention has been paid to the aquatic compartment. Water is the destination of contaminants present in the air (through rainfall, for example) and in the soil (through runoff); furthermore, aquatic organisms, due to their anatomical and functional characteristics, are considered more exposed to the risks deriving from contaminants dissolved in water [1,2,3].

In the past, soil and its inhabitants were considered partially protected from the effects of pollutants, often being able to accumulate them without apparently compromising the survival of organisms. In fact, the first ecotoxicological studies on soil concerned its buffer capacity and bioaccumulation, mainly by plants, often used for the recovery of heavily polluted soils (phytoremediation) [4,5,6,7]. Undoubtedly, the bioaccumulation capacity of plants used as food for animals intended for human consumption has led to an increasing number of studies on the toxic effects of various soil contaminants on model animals, such as rats and mice, in order to monitor any damage resulting from the consumption of contaminated food [8,9]. More recently, following a greater awareness of the importance of protecting biodiversity, studies aimed at identifying the effects of soil contamination on terrestrial animals have increased considerably [10,11]. On the other hand, the application of pesticides and fertilizers aimed at increasing soil productivity and the dumping of waste have worsened soil quality [12,13].

To determine the risk faced by animals exposed to soil contaminants, invertebrates such as earthworms, gastropods, and insects have been extensively studied [14,15]. Their proven ability to biomagnify has demonstrated how environmental contamination can be transferred through the food chain, affecting main soil predators such as reptiles, birds, and mammals [16,17]. Toxicokinetic, toxicodynamic, and cytotoxicity studies in terrestrial vertebrates are mainly carried out on laboratory animals [18,19,20]. Wild species are studied little, except in the field of biomonitoring [21,22]. In recent years, this trend has been reversing, and research results on the toxic effects of various soil contaminants (heavy metals, fertilizers, herbicides, and pesticides) on reptiles and birds are increasingly being published [17,23,24].

**Figure 1:**
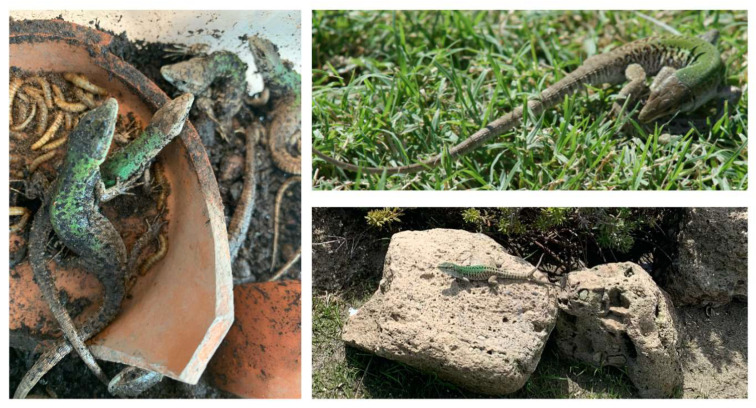
Specimens *of P. siculus* in captivity (**left**) or natural environments (**right**).

This review summarizes the studies performed on the lacertilian reptile of the *Podarcis* genus, a group of resilient wild species capable of living in anthropized areas [25]. Particularly studied is the *P. siculus* lizard (formerly known as *Lacerta sicula* and *P. sicula*; Figure 1), which is widespread throughout the Mediterranean area and beyond [26,27,28]; inhabiting agricultural areas and city parks, it is exposed to the action of contaminants, especially those used in agriculture (fertilizers and pesticides) or deposited on the ground (for example, heavy metals).

Being an animal that can be easily captured in the milder periods of the year (from March to October in Italy) and kept for a long time in a terrarium, *Podarcis* lizard lends itself well to molecular, morphological, and physiological studies; the availability of manipulating freshly laid eggs in captivity has also allowed the identification of the various stages of its embryonic development [29]. The reproductive cycle and gonad organization of *P. siculus* are also well known in both females and males [30,31]. The knowledge of the biology of this reptile represented a good starting point for cytotoxicological studies aimed at identifying the damage induced by the most common soil contaminants on the morphology and functionality of tissues and organs.

The objectives of this review paper were to collect, compare, analyze, and summarize information about pollutants that endangered *Podarcis* lizards, the adverse effects on their tissues and organs, and the role played by different routes and doses of exposure. The review was divided into sections that outlined different groups of pollutants, the type of exposure (environmental or laboratory), and the recorded effects in lizards; the latter were compared with effects found in other animals. Studies carried out on lizards can provide tools to recognize the dangers and risks that threaten terrestrial vertebrates when exposed to environmental pollutants.

## 2. Literature Review

The literature was searched for information on the toxic effects of various contaminant classes, including pesticides (insecticides, fungicides, and herbicides), endocrine-disrupting chemicals (EDCs), and metals, on lizards of the *Podarcis* genus. The study was focused solely on the lacertids of the *Podarcis* genus, as these lizards serve as a valuable reptile model, widely utilized in cytotoxicology and environmental toxicology studies. The search was carried out across various scientific databases (PubMed, Scopus, Web of Science), using *Podarcis* and the aforementioned contaminants as keywords. In detail, “Podarcis” and “toxicity” returned a maximum of 45 articles; “Podarcis” and “pollution” a maximum of 22 articles; “Podarcis” and “pesticide(s)” a maximum of 29 (with fewer articles when using the keywords “herbicide”, “fungicide”, “insecticide”); “Podarcis” and “endocrine disrupting compounds (or chemicals)” 13 articles; “Podarcis” and “metal(s)” 23 articles (22 using the keyword cadmium). The bibliographic search ended in September 2024. A total of 39 articles (excluding redundant titles) met the inclusion criteria; that is, they concerned studies carried out on lacertilian reptiles of the genus *Podarcis*. Half of the works (49%) studied the effects of pesticides, 33% the effects of the heavy metal cadmium, and the remaining 18% the effects of alkylphenols, i.e., chemicals belonging to the EDCs group. All studies were conducted in Europe, most in Italy (76.9%), followed by Portugal (15.4%) and Germany (7.7%). Table 1 lists the retrieved articles, along with the pollutants tested and exposure routes. Only 4 articles (10.25%) tested the effect of cadmium-contaminated soil on the development of *Podarcis* embryos.

## 3. Pesticides

A wide range of chemical compounds is globally used as pesticides to eradicate undesired pests, especially in agriculture [70]. Lizards can be easily exposed to pesticides in several ways, including ingestion of contaminated food, dermal exposure, inhalation, maternal transfer to eggs, and absorption by eggs from surrounding environments [71,72,73]. Among the various substances used in agriculture, *Podarcis* has accidentally come into contact or has been experimentally exposed to fungicides such as methyl-thiophanate [44,45,46,47,48], carbendazim, copper sulfate [32,33,34,35,36,37,38,39]; herbicides (diuron, glyphosate) [39,40,41,42,43], and insecticides (chlorpyrifos, deltamethrin, cyhalothrin) [32,33,34,35,36,37,38,49,50]. Some scientific articles have investigated the effects caused by accidental exposure to pollutants in natural habitats following human activities [32,33,34,35,36,37,39]; many other studies have been carried out on lizards experimentally exposed to one or more contaminants normally present in the environment [29,38,40,41,42,43,44,45,46,47,48,49,50,51,52,53,54,55,56,57,58,59,60,61,62,63,64,65,66,67,68,69]. The data obtained fulfill the dual purpose of evaluating the risk faced by terrestrial vertebrates living in contaminated areas (biodiversity conservation studies) and of transferring information on the toxic effects of contaminants at the cellular and tissue level to other animal models, including humans.

### 3.1. Environmental Exposure

The consequences of living in agricultural areas particularly exposed to mixtures of pesticides and fertilizers were studied at a demographic and morphological level on natural field subpopulations of *Podarcis bocagei*, collected in six geographically similar localities in Portugal. Four sites were under intensive agriculture, and two sites were under organic agriculture. In the first fieldwork [33], the researchers examined parameters in vivo such as population size and density, sex ratio (*P. bocagei* adults show a strong sexual dimorphism) [74], body size, ectoparasites prevalence, and fluctuating asymmetry in femoral pores, a biomarker of environmental stress in lizard [75]. The authors detected a few statistically significant differences between the animals exposed to pesticides and those not exposed. According to the authors, the absence of observable effects among subpopulations living in habitats subjected to different anthropic pressures is indicative that lizards are resilient organisms capable of coping, within certain limits, with adverse stimuli [33].

The authors also investigated biochemical, histological, and physiological differences between the different lizard subpopulations [32]. Evaluated biomarkers included body condition index, standard metabolic rate, locomotor performance, endoparasites, enzymatic activity of oxidative pathways, lipid peroxidation, liver, and testis histology. Animals from less contaminated locations showed better health conditions than individuals from exposed sites, having a greater body mass. No significant differences were recorded in locomotor performance and the level of oxidative stress. A slight increase in standard metabolic rate and blood parasites was found in animals collected from agricultural sites exposed to pesticides. Mild histopathological changes, such as congestion, fibrosis, degeneration, and vacuolization of hepatocytes and iron pigments, were observed in the liver of lizards, regardless of the capture site. Overall, the authors concluded that lizards living in sites exposed to various pesticides have lower ecological suitability than those living on-site treated with organic products. However, the toxicity associated with pesticide exposure did not appear to severely affect the populations [32,33].

Two fieldworks carried out by Mingo et al. also tested body condition and the activity of 3 biomarkers of pesticide exposure in wild populations of the lizards *Podarcis muralis* [34,35]. It is very interesting that, for the first time, the authors performed their analyses on saliva from buccal swabs, thus using a minimally invasive sampling method, which allowed them not to sacrifice the animals previously captured in the studied areas. Four sampling sites in Rhineland-Palatinate, Germany, were considered: three vineyards with different soil use intensities (10% to 70%) and a reference site (0% agricultural use). In the vineyards, a mixture of pesticides was applied by aerial dispersion in different combinations at 7–10-day intervals. Following pesticide applications, the authors recorded the increase in the enzymatic activities of Glutathione Reductase (GR) and Glutathione-S-Transferase (GST), strong indicators of oxidative stress and detoxification processes; no significant differences in Acetylcholinesterase (AChE) activity were detected. Hence, the neurotoxic effects of the applied pesticide formulations were not attested. GR and GST activity increased slightly across exposure gradients, while the fitness of lizards worsened. Moreover, the enzymatic patterns allowed the authors to affirm that the uptake of pesticides occurred both via the skin and orally; according to their conclusions, the initial increase in activity, recorded two days after the application of the pesticides, was caused by skin absorption, followed by a normalization of activity and subsequent new increase due to the delayed effect of oral exposure, since the accumulation of pesticides in the lizards’ prey was lower [34,35].

Since the use of buccal swabs could represent a reliable method for detecting the effects of exposure to toxic substances in terrestrial vertebrates, much less invasive than traditional analysis of blood and organ samples, the same authors repeated the experiments in the laboratory [38]. *P. muralis* lizards captured in a pristine area were divided into control, skin, and oral treatment groups and exposed to different pesticide formulations at conventional field doses. Subsequently, the enzymatic activity was analyzed on buccal swabs. The authors confirmed the suitability of the method to detect the effects of pesticide exposure at the enzymatic level, finding a remarkable similarity between field and laboratory results. Interestingly, another study [76] compared the enzymatic activities of saliva, organ, and blood serum samples of the Asian lizard *Eremias argus,* and correlations were found between different samples, confirming the buccal swab as a useful non-invasive method in reptiles.

In another field research [39], the health status of the thyroid gland and testis was compared in *Podarcis bocagei* lizards collected in different areas: in two fields used for over 30 years for the cultivation of corn and where a mixture of pesticides was routinely applied, and in two organic agricultural fields with no history of pesticide application. In *Podarcis,* the thyroid shows the typical structure with follicles connected by interfollicular connective tissue containing blood vessels. The analysis of the soils of the corn cultivation fields demonstrated the presence in these soils of 7 different pesticides, having different target organisms and mechanisms of action. Investigations on lizards demonstrated that the thyroids of all individuals had a normal histological appearance; however, differences in thyroid function have been observed. In lizards from sites exposed to the pesticide mixture, many resorption vesicles in the follicular colloid and a larger follicular area were found, while no differences in thyrocyte height were detected [39].

As regards male gonads, *Podarcis* testis is made up of seminiferous tubules morphologically and functionally, like those of birds and mammals. During the breeding season, the tubules consist of germ cells in all stages of differentiation with numerous spermatozoa, ready to be ejaculated. In the resting period, the seminiferous tubules are composed of Sertoli cells and a few germ cells, mostly spermatogonia [43]. Histological examination of testes showed that most lizards from the pesticide-free sites had only two spermatogenesis layers (spermatogonia and spermatocytes I), consistent with the lizards’ capture period (November); pesticide-exposed lizards revealed the presence of all spermatogenesis layers, including spermatozoa. The result indicates that lizards in contact with contaminated soils were in a more advanced stage of spermatogenic activity, showing an anticipation of the differentiation of spermatogonia into more mature germ cells compared to the natural period of the reproductive cycle. No differences in circulating testosterone concentrations were found between animals, while immunohistochemical analysis demonstrated a different expression and localization of thyroid receptors: in lizards from pesticide-treated sites, the signal was detected in some immature germ cells and all mature spermatozoa; in pesticide-free sites, positive signal was only in Leydig cells [39]. These findings suggest that environmental exposure of lizards to a mixture of pesticides dysregulates endocrine activity on the thyroid gland, which, in turn, may affect the male reproductive system. These results agree with those observed in mammals, where alachlor, one of the pesticides in the mixture, has been shown to impair thyroid function [77,78]. In their conclusions, the authors highlight the risk faced by terrestrial vertebrates living near fields dedicated to conventional agriculture.

Another series of environmental exposure analyses investigated the effects of different mixtures of pesticides commonly used for hazelnut orchards on *P. siculus* lizards inhabiting these areas [36,37]. The health status of captured wild lizards was studied using a multi-biomarker approach and compared with that of lizards living in a pesticide-free area. The authors analyzed the ability of lizard tissues to accumulate and metabolize the different compounds, the activities of the enzymes AChE and GST, the production of free radicals, the frequency of micronuclei, and, finally, the onset of parasitic infections. The collected findings showed how conventional treatments in agriculture affect the wild animals that live and feed in those areas. The results highlighted a partial accumulation of several chemicals, the involvement of defense mechanisms, and some cellular damage after exposure to environmental pesticides [37]. Conventional treatments induced damage to lipids and the formation of hydroxyl radicals, to which cells responded by increasing their antioxidant machinery; nevertheless, DNA damage was found, but not neurotoxicity. No difference was also detected regarding intestinal parasite infections.

Overall, the fieldwork data demonstrate that lizards are good bioindicators of soil conditions, further legitimizing the laboratory studies carried out over the years that have used this lizard as an alternative model organism to mammals such as rats and mice.

### 3.2. Laboratory Exposure

#### 3.2.1. Methyl Thiophanate

Methyl thiophanate (Mt) is a member of the thiophanate derivatives possessing a broader range of activity, widely used to control important fungal diseases of crops. In target organisms, once absorbed, it interferes with microtubule function, impairing tubulin polymerization during cell division, thus affecting fungal growth [79]. In animals, ingested Mt is well distributed throughout the body and is metabolized to benzimidazole compounds, including carbendazim (methyl-2-benzimid- azole carbamate), a well-known reproductive toxicant [79,80]. Data on the toxic effects of Mt on non-target organisms are conflicting; some works highlighted the low toxicity of the fungicide even at high doses, while others demonstrated a certain toxicity of Mt, suggesting an endocrine-disrupting effect [81]. For this reason, the first studies carried out using *Podarcis* as a model investigated the effect of Mt on the adrenal glands and thyroid [45,46,47,48].

In the work of De Falco et al. [45], the condition of the adrenal gland of adult *P. siculus* specimens housed for 15 or 30 days in terrariums containing soil, food, and water polluted with 1.5% Mt was studied to simulate environmental exposure to the fungicide. The 1.5% Mt solution corresponds to the concentration of Mt sprayed on crops fruit and ornamental plants.

In lizards, the adrenal glands comprise both steroidogenic and chromaffin tissues in contact with the gonads and genital ducts [82]. The authors demonstrated that Mt negatively influenced the normal function of the adrenal glands by affecting the balance of hormone levels, mimicking endogenous hormone function, and altering histo-morphological features of the gland [45]. A time-dependent increase in plasma levels of corticosterone and adrenaline, a decrease in plasma levels of adrenocorticotropic hormone (ACTH) and noradrenaline, hypertrophy of the steroidogenic tissue, and an enlargement of blood capillaries were observed, together with infiltrations of lymphocyte and macrophage, typical signs of tissue degeneration [83].

This kind of “forced” environmental exposure has been used to establish the onset of other adverse effects of Mt on *P. siculus* [44]. In the liver, Mt exposure led to sinusoid dilation, glycogen depletion, increased apoptosis, and peroxisomal proliferation, mediated by increased expression of peroxisome proliferators activated receptors (PPARs). The authors conclude that even in terrestrial ectotherm vertebrates, where energy metabolism is quite different from homeotherms, PPARs can be used as biomarkers in ecotoxicological studies to evaluate pollution of terrestrial environments [44].

Other research investigated the genotoxic effects of this exposure using the micronucleus test, the analysis of chromosomal aberration and instability, and the comet assay as markers [47]. After exposure to Mt for 30 and 40 days, the number of micronuclei in erythrocytes increased significantly; DNA damage, assessed by comet assay on the same cells, documented a significant increase in comet length in relation to exposure time; cytogenetic analysis carried out on spleen and bone marrow tissues showed a significant increase in chromosomal aberrations in exposed animals.

For the study aimed at evaluating the effects of the fungicide on the thyroid gland [46], Mt was administered intraperitoneally to *P. siculus* males at different concentrations in single or multiple injections. Dose-dependent mortality was recorded, with up to 70% (acute exposure) and 20% (chronic exposure) of animals dying at the highest Mt concentrations. Mt-treated lizards presented dose-dependent morphological changes in the thyroid gland, leading to poor functional activity. The animals also showed reduced plasma T4 and T3 levels compared to control animals, as well as reduced serum TSH levels, thus suggesting adverse effects of Mt also on the pituitary gland [46]. Overall, the data collected from the adrenal gland and thyroid experiments strengthen the case for the fungicide Mt as an endocrine disruptor.

The data collected by Cardone [48] on the testis of *P. siculus* also point in this direction. Severe morphological and physiological changes occurred in the testes of Mt-treated animals, both after acute and chronic exposures. The decrease in the expression of androgen and estrogen receptor mRNAs was recorded in all experimental groups correlated to exposure time. The disorganization of the wall of the seminiferous tubules with evident signs of apoptosis, the vacuolization of Sertoli cells, the presence of multinucleated giant cells, the decrease in germ cells with complete loss of spermatozoa clearly demonstrated the impairment of lizard spermatogenesis following Mt-treatment, probably due to the lack of expression of sex steroid hormone receptors, as postulated for mammals [84]. Crucially, all reproductive endpoints in MT-treated lizards were affected, suggesting that uncontrolled exposure to this fungicide could induce infertility in male lizards.

#### 3.2.2. Chlorpyrifos

Chlorpyrifos is an organophosphorus insecticide widespread throughout the world for many years; used since 1965, its use was banned in Europe only in 2020, once its neurotoxicity and genotoxicity in humans had been ascertained. Indeed, like other chemical compounds used in agriculture, it represented a risk for terrestrial organisms inhabiting the treated soils and, through trophic transfer, for all animals [85].

Amaral et al. investigated the effects of dietary exposure to sublethal concentrations of chlorpyrifos on the lizard *P. bocagei* [49]. The animals were fed with live mealworms contaminated with two different concentrations of insecticide, corresponding to the residual concentrations of chlorpyrifos reported in mealworms [86]. After exposure, the response of various biomarkers was evaluated in different tissues, along with the onset of hepatic and testicular histopathologies and changes in locomotor and predatory behavior. The authors stated that this dietary exposure did not cause any significant changes in the activity of antioxidant enzymes, lipid peroxidation, and glutathione in the different tissues examined; conversely, carboxylesterases and cholinesterase activities decreased in a dose-dependent manner. The authors also described severe hepatic histological changes in animals exposed to chlorpyrifos, such as fibrosis, hepatocyte degeneration, and vacuolation. No toxic effects on testicular morphology and function have been noted, regardless of the exposure dose. Finally, after exposure to chlorpyrifos, animals took longer to capture and swallow prey [49]. The authors hypothesized that this issue could be explained by the observed inhibition of cholinesterases, which, in turn, could cause an accumulation of acetylcholine in synaptic junctions, thus altering neuromuscular capabilities and decreasing muscle coordination essential for prey manipulation. The latter chlorpyrifos-induced feature could be the most harmful since if exposure to pesticides results in behavioral alterations, the lizards’ growth, reproductive success, and survival could be affected.

#### 3.2.3. Diuron

Diuron [3-(3,4-dichlorophenyl)-1,1-dimethylurea] is an herbicide derived from phenylurea [87]; absorbed by the roots of the plant, it inhibits the photosynthetic process [87]. Long used to control weeds in agricultural crops such as fruit, cotton, wheat, and soybeans, the European Union revoked its use in 2022 due to its persistence and bioaccumulation in soil, sediments, and water [88,89].

Diuron toxicity has been studied in soil microbiota and soil organisms such as worms and snails; more recently, Cardone et al. [40] studied the effects of diuron on the reproductive system of adult males of *P. siculus*. Although no animal deaths were recorded during experimental treatment with diuron, the authors described severe testicular effects of greater magnitude in lizards exposed to contaminated water. The gonadosomatic index decreased significantly, and the seminiferous tubules were markedly reduced in the cross-sectional area due to the collapse of the seminiferous epithelium. In the most severely damaged tubules, only Sertoli cells and some spermatogonia were present, with the complete loss of all stages of germ cell maturation. The interstitial spaces appeared hypertrophic and contained numerous lymphocytes, neutrophils, and monocytes, typical markers of inflammation and necrosis. The epididymis also appeared to have regressed with abundant connective tissue, while the epithelial cells did not present secretory granules. Finally, in treated animals, testosterone levels decreased significantly, as did estrogen, which was undetectable.

There is no doubt that the results described by Cardone et al. [40] indicate that diuron in exposed lizards caused male reproductive toxicity. Interestingly, research by Fernandes et al. [90] showed a lack of reproductive toxicity induced directly by diuron in male rats. This finding highlights the importance of ecotoxicological studies in evaluating the harmful effects of environmental contaminants directly on the organisms living in the polluted environment rather than using surrogate species, as often happens for reptiles and birds, which are equated with mammals [11]. Although tissue morphology may appear similar between reptiles, birds and mammals, the different metabolism and detoxifying capacity of some organs can have a significant impact, leading to erroneous conclusions, which may endanger the survival of wild species.

#### 3.2.4. Glyphosate

Glyphosate (N-(phosphonomethyl) glycine, Gly) is an organophosphate, non-selective herbicide widely used for agricultural weed control [91]. By inhibiting the biosynthesis of aromatic amino acids in plants, i.e., a pathway absent in animals, Gly has long been considered safe for animals [92]. However, the accumulation of Gly and its main metabolite, the aminomethylphosphonic acid [93], has led the scientific community to question its effective harmlessness; furthermore, detection of the herbicide in human urine demonstrated its absorption through the food chain [94]. From these results, a series of studies began on the experimental administration of Gly to various invertebrates and vertebrates to verify its effects on non-target organisms [95].

The effect of Gly on the lizard *P. siculus* was recently studied; for these studies, sexually mature lizards were exposed to glyphosate by oral administration [41,42,43,96,97]. Overall, the results gathered from these studies demonstrate that Gly, albeit administered at low doses, affects the lizard’s liver and gonads, inducing many morphological and molecular alterations and also acting as an endocrine disruptor.

In females, a significant impact of Gly on ovarian structure has been demonstrated, with a dose-dependent effect [43]. In *Podarcis,* the ovary has a clustered structure, with follicles at different stages of maturation [30]. Immature primary follicles show oocytes surrounded by a monolayer of small stem cells; later, the stem cells proliferate, and those close to the oocyte increase in size and change shape, becoming Pyriform cells [98], i.e., nurse cells that undergo apoptosis before vitellogenesis [99].

The results demonstrated that the herbicide induced the recruitment of primary oocytes and the formation of primary follicles; at the same time, it promoted the apoptotic regression of Pyriform cells, anticipating the maturation of the follicles. It also induced collagen deposits in the follicular epithelium and modified the oocyte cytoplasm and the composition of the zona pellucida, whose proteins showed changes in carbohydrates. The authors also demonstrated that Gly induced the expression of estrogen receptors, also modifying their localization in follicular cells. These changes suggest that the herbicide has endocrine-disrupting effects, as already found in other organisms and cells, and is therefore classified as an endocrine-disrupting compound [100].

Like *P. siculus* females, males also showed alterations in spermatogenesis following Gly treatments [42,101]. Morphological data showed a dose-dependent reduction in spermatozoa production, many empty spaces, aggregates of immature germ cells in the epithelium of the seminiferous tubules, and a huge deposition of collagen fibers in the interstitial spaces between the tubules. The authors also demonstrated a dose-dependent reduction in connexin 43, a gap-junction protein responsible for the crosstalk between Sertoli cells and germ cells [102]; no changes in the detection of steroidogenic enzymes were observed, suggesting no changes in sex hormone levels. As in the female lizards, the herbicide increased the expression of estrogen receptors, accompanied by a broader distribution of ERs in both Sertoli and germ cells.

Finally, these studies also confirmed that Gly is toxic to the liver of these animals, regardless of sex [41,96]. In treated animals, hepatocytes showed a dose-dependent accumulation of lipofuscin and glycogen granules; the liver parenchyma was characterized by swelling of the blood vessels and bile ducts. Abundant connective fibers were also present in the hepatic tissue, forming in the most severe cases of nodular formations, typical of the onset of many liver diseases [103]. The authors confirmed the xenoestrogenic activity of Gly by demonstrating upregulation of the expression of estrogen receptor α and vitellogenin genes in hepatocytes of treated male lizards [41].

Altogether, the data obtained by exposing *P. siculus* to Gly allow for greater awareness of the risks faced by vertebrates living in Gly-treated areas. The toxic effects induced by the herbicide are similar in both female and male gonads and in the liver, an organ strongly involved in the reproductive process of oviparous vertebrates. Reduced adult fecundity could lead to a rapid decline in the number of *Podarcis* individuals and many other vertebrates that share structural and functional characteristics of these organs with the lizard.

## 4. Endocrine Disrupting Compounds

An Endocrine Disrupter Compound (EDC) is an exogenous substance or a mixture that alters the function(s) of the endocrine system, causing adverse health effects in an intact organism, its progeny, or (sub)populations [104].

The primary source of EDCs is industry (pharmaceuticals, plastics, flame retardants and preservatives) and agriculture (pesticides, fertilizers), so living organisms can be constantly exposed to them [105]. They affect the endocrine system by preventing the synthesis and function of natural hormones, interacting with hormone receptors, acting as agonists or antagonists, and influencing hormone transport. The molecular structures of EDCs make them like hormones such as T3, T4, and estrogen, thus mimicking their action [105]. The increase in infertility and obesity observed in the human population in recent years is often related to exposure to EDCs [106,107]. EDCs present in the environment include bisphenyls, organophosphates, and phthalates.

### 4.1. Polychlorinated Biphenyls

Polychlorinated biphenyls (PCBs) are a group of industrial chemicals produced by the chlorination of biphenyl, leading to complex mixtures of congeners. Initially produced and used worldwide in large quantities, they were banned in the late 1970s due to their adverse effects on living organisms and the environment caused by their persistence, bioaccumulation, and toxicity [108]. Unfortunately, although no longer produced, they represent contaminants of concern; major primary sources of PCBs into the environment include poor disposal practices, volatilization and runoff from landfills containing PCB waste, and incineration of waste containing PCBs [109].

They are classified as EDCs because they can interact and interfere with different functions of the endocrine system [110]; similar in structure to thyroid hormones, they severely affect the thyroid gland [108].

A recent study [57] investigated the relationship between PCB-contaminated soils and the effects on *P. siculus* lizards in terms of accumulation, interference with the hypothalamic–pituitary–thyroid axis, and morpho-physiology of the thyroid gland. Adult males were captured in May, when the thyroid gland was in full functional activity [111], and housed in terrariums containing soil contaminated with different concentrations of a mixture of naturally occurring PCB congeners. Determination of PCB mixture in plasma and tissues demonstrated that lizards absorbed PCBs through skin penetration when living in contaminated soil. The concentration of PCBs, when absorbed by the soil, was skin > plasma > liver > kidney > brain; in PCB-injected lizards, the lowest concentration of PCBs was found in the skin, followed by brain < kidney < plasma < liver [57].

A significant dose-dependent increase in mortality and decrease in body weight was observed in animals; since no decrease in appetite was recorded, the authors linked the weight reduction with alterations in thyroid function. A dose-dependent increase in TRH levels and a decrease in TSH levels were recorded, and T3 and T4 values were also drastically reduced in both plasma and hepatocytes. The morphological analysis of the thyroid gland in animals housed in PCB-contaminated terrariums showed the thyrocytes constituting the follicular epithelium reduced in height, with small and elongated nuclei with dense chromatin and little cytoplasm, a retracted colloid with very few reabsorption vacuoles. Overall, the results indicated that PCBs affected thyroid homeostasis and morphology, no matter the route of exposure, which, on the contrary, influenced their accumulation in the different lizard tissues.

### 4.2. Alkylphenols

Alkylphenols are a family of organic compounds obtained by the alkylation of phenols. Ethoxylated alkylphenols are widely used as industrial surfactants; they include commercially relevant propylphenol, butylphenol, amylphenol, heptylphenol, dodecylphenol, nonylphenol (NP) and octylphenol (OP) [112]. The last two are also the main degradation products of all alkylphenols [113]. Since they are not produced naturally, their presence in the environment is a consequence of anthropogenic activity; they enter the environment primarily through industrial and municipal wastewater treatments, direct discharge, and pesticide application. NP and OP have been detected in air, water, soil, sediments, and biota [112].

Alkylphenoles are classified as EDCs; they exert xenoestrogenic feminizing effects in vertebrates, resulting in impaired reproduction [114,115]. It has been demonstrated that NP, for example, can act like the female hormone 17β-estradiol by binding to the estrogen receptor and competitively displacing 17β-estradiol [116].

As with other substances released into the environment, especially through water, most of the studies on the toxic effects of alkylphenols and EDCs in general have concerned aquatic species [117,118]. Subsequently, detecting these substances in the food web and the soils also extended the studies to soil inhabitants [119]. On the other hand, it must be considered that many alkylphenols are part of the composition of pesticides as additives [120].

As regards *Podarcis* lizards, four scientific publications describe studies carried out on *P. siculus*; this research involved the experimental exposure of animals to NP and OP. A first study demonstrated that NP, administered via food and water, was able to induce the expression of both vitellogenin and ERα in the male liver of *P. siculus*; vitellogenin, undetectable in untreated males, rapidly accumulated in plasma after administration of the NP-polluted diet [51]. The finding of plasma vitellogenin in male oviparous vertebrates is indicative of xenoestrogen contamination [121,122], and this demonstrated the endocrine-disrupting activity of NP also in lizards.

The study by De Falco et al. [53] investigated the effects of NP on the hypothalamic–pituitary–adrenal gland (HPA) axis. Lizards of both sexes received intraperitoneal injections of NP for periods of 12 to 76 days. The authors found a time-dependent stimulation of the HPA axis, with increased plasma levels of the hypothalamic CRH, the pituitary ACTH, and the adrenal hormone corticosterone; plasma catecholamine concentrations showed a different response, with the increase in adrenaline and a concomitant decrease in noradrenaline plasma levels. Morphological analysis showed enlargement of blood capillaries and vascularization, hypertrophy of steroidogenic cords with vacuole-rich cells, increased number of adrenaline-secreting cells, and decreased number of noradrenaline-secreting cells. Unlike steroidogenic cells, chromaffin cells showed a general decrease in secretory granules. The authors also described strong lymphocyte infiltration with a large number of macrophages. These data indicate stimulation and concomitant inflammation of adrenal glands following NP exposure. The authors reported no gender-related differences.

Similar results were retrieved on *P. siculus* lizards exposed to OP alone or both the alkylphenols OP and NP [54]. The alterations in the plasma levels of CRH, ACTH, corticosterol, and catecholamines were even more evident with the combined treatment, as were the morphological alterations affecting the gland, which at the end of the treatment was particularly hypertrophic and inflamed. Interestingly, a recovery period of 15 days from the last injection, regardless of the injected solution (OP alone or OP + NP), restored plasma hormone levels, whereas the morphology of the gland was still altered. It is conceivable that a more prolonged recovery period was necessary to ameliorate the gland’s condition to bring it back to morphological control conditions.

Similar studies [56,123] have been carried out to evaluate the response of the hypothalamus–pituitary–thyroid axis to NP and/or OP. The thyroid gland of NP-treated lizards showed dose-dependent morphological changes; the follicular epithelial cells were reduced in height, and the thyrocytes had small and elongated nuclei with dense chromatin and a very reduced cytoplasm. The colloid was retracted with a few resorption vacuoles. Plasma concentrations of TSH, T4, and T3 decreased in a dose-dependent manner [123].

The animals exposed to the two alkylphenols showed a reduction in the volume of the thyroid follicles; the morphology of the epithelial cells resembled that observed in NP-treated lizards, typical of non-functionally active thyrocytes. Moreover, both treatments induced an increase in plasma TRH levels and, conversely, a decrease in TSH, T3, and T4 levels. The liver of treated lizards also showed a higher concentration of T3 and a lower concentration of T4, probably caused by an increased activity of iodothyronine 5′-deiodinase type II enzyme [56]. Collectively, the data suggest a severe interference of OP and NP with the hypothalamus–pituitary–thyroid axis and a systemic imbalance of thyroid hormones, with changes in their bioavailability and metabolism in peripheral organs.

Finally, the effects of OP and NP, alone and in mixture, were evaluated in *P. siculus* testis during the reproductive period [55], considering the strong xenoestrogenic action exerted by these compounds [52]. The treatments altered testicular morphology with a more preponderant detrimental effect of OP than NP and in a dose-dependent manner. After OP exposure, the final stages of differentiation (spermatids and spermatozoa) disappeared, the diameter of the seminiferous tubule and the thickness of germinative epithelium decreased, while the intertubular connective tissue increased. In NP-treated lizards, it was still possible to highlight the presence of germ cells in all stages of differentiation, even if only a few spermatozoa were detectable in the particularly large lumen of the tubules. Interestingly, all treatments did not cause significant alterations in somatic Sertoli and Leydig cells, which appeared distributed as in the testes of untreated animals [55]. The authors also evaluated the changes in the localization of the steroidogenic enzymes 3β-hydroxysteroid dehydrogenase (3β-HSD), 17β-hydroxysteroid dehydrogenase (17β-HSD), and P450 aromatase, key enzymes in the synthesis of testosterone [124] and 17β-estradiol [125,126]. Under natural conditions, in male lizards, steroidogenic enzymes are recorded in both somatic and germ cells during reproductive periods, with some exceptions: P450 aromatase is abundant in mature germ cells and Leydig cells, while it is scarce in spermatogonia and Sertoli cells and is absent in spermatocyte [124,125]. NP and OP alone or in a mixture altered the localization of these enzymes; in particular, the authors described a general decrease in the cellular localization of both 3βHSD and 17βHSD and a more marked inhibition of P450 aromatase, completely absent in all germ cells. The reduction in the enzymes involved in the production of sex hormones operated by APs, particularly OP, affected spermatogenesis and spermiohistogenesis, thus explaining the absence of spermatids and spermatozoa recorded by the morphological analysis [55].

## 5. Potentially Toxic Elements

Among the potentially toxic elements, cadmium (Cd) is the one whose effects have been studied on lizards. Cadmium is a non-essential trace element belonging to the heavy metals, metallic elements with high density and high atomic number, present throughout the earth’s crust; they are also called trace elements due to their presence in concentrations lower than 10 ppm in organisms and various environmental matrices [127]. Some of them are defined as essential trace elements because they exert biochemical and physiological functions in plants and animals, constituting several key enzymes; zinc, copper, and iron are the most abundant and important in living organisms [128]. Other metals such as aluminum, arsenic, cadmium, lead, and mercury have no established biological functions and therefore are considered non-essential, potentially toxic elements [128,129]. The latter is particularly toxic, even at very low concentrations, due to their ability to displace other essential metals from biological ligands; however, it must be considered that the essential metals themselves can be toxic when their cellular concentration increases [128,130]. The multiple applications of metals, both essential and non-essential, have led to their wide distribution in the environment. Their toxicity depends on the dose, route of exposure, and chemical species [129,131]. Cd is a priority contaminant due to widespread environmental contamination; the main anthropogenic sources are atmospheric emissions resulting from metal smelting and burning of fossil fuels, modifications of agricultural soils with Cd-contaminated fertilizers, and industrial waste. Cd accumulates easily in vegetables, thus entering the food chain; its biological half-life of approximately 30 years greatly increases its potential toxicity [129,132]. In terrestrial vertebrates, exposure to Cd usually occurs because of ingestion [133]; once in the digestive tract, the ion can cross the luminal membrane via various transporters that normally transport other essential metals, such as zinc and calcium and forms stable S-conjugates with low molecular weight thiols such as glutathione or binds cysteine-rich proteins such as metallothionein [134]. Cd has been shown to induce toxicity in the lungs, liver, kidneys, testes, and brain; it is also classified as a known human carcinogen based on experimental studies showing an association between exposure and incidence of cancer in humans and animals [132].

### 5.1. Studies on the Bioaccumulation of Cadmium in Podarcis Tissues

The first studies aimed at investigating the toxic effects of cadmium on field lizards addressed the toxicokinetic of the metal when ingested. Mann et al. [59,60] exposed adult individuals of *Podarcis carbonelli* to dietary Cd for 21 weeks. As expected, the highest accumulation levels were recorded in the intestine, and higher rates of assimilation were observed over the first five weeks; a slower redistribution to other tissues was then recorded. The authors found that Cd accumulated in tissues in the following concentration order: intestine > liver > kidney > carcass, thus suggesting the passage of Cd through the intestine into the bloodstream. However, no changes in biomarkers of metals exposure, such as metallothionein (MT) content, were observed [59,60].

The research of Trinchella et al. [58] also studied the accumulation and distribution of Cd in different organs of the lizard *P. siculus* under different routes of exposure; in addition, the authors evaluated the relationship between Cd accumulation and MT gene expression in lizard organs. For the treatment simulating acute Cd exposure, adult animals received a single intraperitoneal injection of a Cd solution; to simulate chronic dietary treatment, lizards were orally administered Cd every two days for 60 days. The authors stated that neither treatment resulted in mortality. After acute exposure, differences in the Cd content were observed, mainly in the kidneys and ovaries, with Cd levels 250 and 125 times higher than in control groups, respectively; these two organs showed, however, a high level of Cd clearance, as their concentration returned to the control level 7 days after treatment. In contrast, in the liver, the Cd concentration gradually increased over the days; the authors hypothesized that the liver could sequester Cd ions released by the kidneys and ovaries. In the brain, a slight but significant Cd accumulation was detected without clearance. This type of exposure induced *MT* gene expression in the liver, kidneys, and ovaries; no increase in *MT* expression was observed in the brain [58].

In agreement with the data collected by Mann et al. [59,60], after oral administration of Cd, the first site of accumulation in *P. siculus* was the intestine, and then the ion reached other tissues, accumulating with the concentration order: intestine > liver > kidney > ovary > brain. Overall, Cd continued to accumulate throughout the 60 days of the experiment. Regarding *MT* gene expression, when lizards were orally exposed to Cd, the amount of *MT* transcript greatly increased in the intestine after 30 days of treatment; after 60 days of treatment, *MT* mRNAs increased in the ovaries and kidneys, while no induction of the *MT* gene was recorded in either the brain or in the liver throughout the treatment, although in the latter Cd accumulated significantly. For this very reason, the authors stated that, in the experimental conditions analyzed, it is not possible to establish a clear relationship between metal accumulation and *MT* gene induction, at least not for all tissues, but depending on the organ and the route of administration of the metal [58].

### 5.2. Cadmium Toxicity Studies on Podarcis Tissues

As with other environmental contaminants, the first studies regarding the toxic effects of Cd on lizard cells and tissues concerned the adverse effects of Cd on the endocrine system, in this case, the pituitary gland.

Ferrandino et al. investigated the toxic effects of Cd ions on the *P. siculus* pituitary gland after acute [61] or chronic [62] treatments.

For acute treatment, the authors found that the morphology of the hypophysis did not change, but they demonstrated an increase in apoptosis, particularly in the lactotroph cells [61]. For chronic treatment, the authors demonstrated the cytotoxic action of the ion, operated at both morphological and physiological levels. The gland showed time-dependent tissue disorganization, with the appearance of atrophied areas, large intercellular spaces, and an increase in vascular network. At the physiological level, the pituitary gland showed an increase in cells producing prolactin and the adrenocorticotropic hormone, which accumulated cytoplasmic granules, indicative of a hormonal accumulation in these cells, probably due to the inhibitory effect of Cd on the release of hormones in the bloodstream. Interestingly, at the end of the treatment (120 days), the number and condition of these cells returned to values like those observed in control glands. The authors hypothesized an adaptive mechanism of the cells favored by the probable activation of defense mechanisms, such as the induction of MT [62].

On the *P. siculus* lizards exposed to a single intraperitoneal exposure of Cd, Favorito et al. [65] investigated the state of the brain, using histological staining and immunological detection of glial fibrillary acidic protein (GFAP), the main marker of astroglial cells [135]. The *P. siculus* brain is characterized by a homogeneous and well-vascularized tissue; neurons and glial cells show a regular shape and a well-defined edge; GFAP immunopositive cells are present in the telencephalon parenchyma, optic tectum, and medulla oblongata [65]. Whereas no changes were observed in the brains of lizards 2 days after treatment, clear morphological alterations were observed at 7 and 16 days after the single injection, as well as the decrease in GFAP immunopositive cells, particularly in the telencephalon, optic tectum, and in the hindbrain. A widespread increase in edema was also observed; the authors speculate that Cd can damage the integrity and functions of the blood–brain barrier since it has been proven that the alteration of this structure is the main cause of the formation of edema [136].

An animal organ on which the toxic effects of Cd have been extensively studied is the liver due to its role as a detoxifier and primary barrier to contaminants present in the bloodstream [137]. The lizard *P. siculus* is no exception, and in 2010, Simoniello et al. verified any liver damage caused by Cd introduced into animals with different doses and administration routes [29]. Although no mortality or alteration of animal behavior was recorded during the experiments, the authors described many molecular and morphological alterations of the liver parenchyma. Cd accumulated in the parenchyma with a half-life of approximately 8 days in single-dose treated specimens and continued to accumulate in long-term dietary treatments. The treated samples showed altered morphology starting from day 2, even in the single food dose. It is interesting to note that the hepatic alterations recorded were similar, regardless of the route and duration of exposure [29]. The authors found extensive edema, enlargement of the sinusoids, and infiltration of erythrocytes into the parenchyma; the hepatocytes showed marked hydropic swelling and alteration of lipid and sugar metabolism. Cd administration induced the *MT* gene expression, as in the liver of untreated animals, exclusively in Kupffer cells and monocytes, which, however, were both increased in number, although no cell proliferation or apoptosis was found [29]. This finding suggests that the lizard liver, when needed, recruits new monocytes that are locally differentiated in Kupffer cells, as occurs in mammals [138]. In conclusion, this research highlights the hepatic toxicity of Cd; the ion reaches the liver even if administered orally and at low concentrations. The organ response is not dose-dependent, and there is no direct correlation between the amount of Cd accumulated and *MT* expression [29]. As already reported in the study by Trinchella et al. [58], *P. siculus* is a good indicator of metal contamination without, however, providing an accurate sign of the number of contaminants present in the environment.

The reproductive toxicity of Cd in *P. siculus* females exposed to the metal via food was also investigated [63,64]. The animals were treated during the breeding season. Cd administration stimulated the recruitment of oocytes from the oogonial pool; the number of primary follicles remained unchanged, while follicular atresia increased, leading to a decrease in the number of growing follicles. Cd also changed the morphology of ovarian follicles, including oocytes. In untreated animals, the growing oocytes were round and surrounded by a thick epithelium; a well-formed zona pellucida was recognizable; both single and multiple Cd-treatments induced comparable effects, such as hydropic swelling of oocytes that showed cytoplasmic vesicles containing clear or filamentous material and many small granules positive on PAS staining. Cd-treated follicles showed increased vascularization of the theca and apoptotic cells, epithelial thickening with protrusions into the cytoplasm of the oocyte, appearance of atresia among the early previtellogenic follicles; the zona pellucida was also affected, appearing disorganized in some places. The various effects induced by Cd on the lizard ovaries led the authors to conclude that the metal mimics the effect of the pituitary follicle-stimulating hormone (FSH) and that Cd therefore acts as an endocrine disruptor. Cd administration significantly reduced the number of eggs laid; multiple Cd treatments were more effective. Embryonic mortality also increased significantly, and the few surviving embryos showed severe deformities, mostly incompatible with postnatal survival [63,64]. Overall, it can be stated that Cd compromises the reproductive fitness of *P. siculus* females by acting on multiple levels: it damages follicles and oocytes, as well as the zona pellucida, thus inhibiting the correct sperm-oocyte interaction, reducing the size of the egg clutch, and causing malformations and death of embryos.

### 5.3. Cadmium Toxicity Studies on Podarcis Embryos

In reptiles, maternal transfer of pollutants to eggs is not the only way to contaminate embryos. Indeed, metal ions and organic contaminants present in soil can pass through the parchment-like shell that characterizes reptile eggs [71,139]. This happens because, at oviposition, the eggs contain insufficient water to complete development, which is absorbed from the soil. It has been proved that *Podarcis* eggs are no exception: when newly fertilized eggs were incubated in Cd-contaminated soil, the developing embryos showed clear morphological [67,68,140] and molecular [69,140] alterations; however, the metal, at the concentration used for these experiments, did not lead to the death of the embryos which continued to develop.

Cd-exposed embryos showed anomalies in the forebrain, midbrain, and eyes; the most frequent malformations were microphthalmia, anencephaly, exencephaly, and various degrees of midfacial hypoplasia, together with the occasional lack of the cranial vault and various deformations of the skull base, palate, and jaws. Cd caused swelling of the diencephalic and mesencephalic ventricles, hyperproliferation, and folding of the retina, while no defects were observed in the medulla oblongata, trunk organs, somites, and limbs. It was also shown that Cd significantly interfered with embryonic gene expression, both activating and blocking the expression of some genes and modifying the levels of transcripts and/or the site of expression of others. Cd-treated embryos exhibited increased expression of the *Otx2* and *Pax6* genes, key regulators of retinal differentiation [141], while their cellular localization remained unchanged [68]. The authors suggested that the increased expression of *Pax6* and *Otx2* could be attributed to the hyperproliferation of retinal cells and not to a Cd-induced overexpression of the two genes. The *MT* gene expression was stronger in Cd-contaminated embryos, particularly in the developing liver and intestine, and not in the brain and eyes, the organs damaged by Cd, where the spatial and temporal localization of *MT* transcripts remained unchanged between untreated and treated embryos [66,67]. In this case, the authors hypothesized a possible link between the absence of induction and the observed embryonic anomalies.

By comparing mRNA expression patterns between control and Cd-treated embryos, Trinchella et al. [69] identified nine upregulated and five downregulated genes. The functions performed by the proteins corresponding to these Cd-responsive genes suggested that many fundamental cellular pathways, such as membrane trafficking, protein–protein interactions, neuronal transmission, and gene regulation, could be affected by Cd exposure; the authors, in particular, underlined how Cd, by modifying the concentration of transcription factors and other proteins capable of regulating gene expression, could exert its toxicity by triggering cascade events which, in turn, involved the expression of many other genes not directly responsive to cadmium.

## 6. Concluding Remarks

Studies performed in recent decades using lizards of the *Podarcis* genus as model organisms have made it possible to verify the absorption, accumulation, and toxicity of soil pollutants on terrestrial vertebrates.

The various aspects investigated concerned the distribution and accumulation of various contaminants (pesticides, EDCs, and heavy metals), the histological descriptions of the effects of these substances on the main endocrine glands (pituitary, thyroid, and adrenals), internal organs (brain, liver), on the reproductive system and embryonic development, as well as on the gene and protein expression in adults and embryos.

These studies provide important contributions to the understanding of the risks related to environmental pollution in non-mammalian vertebrates. Research reveals that many of the effects recorded in lizard tissues at histological, biochemical, and molecular levels are independent of the chemical composition of the contaminants, being mostly linked to the type of cellular response. Table 2 lists the most common effects observed in *Podarcis* lizard following occasional or experimental exposure to various environmental contaminants.

Particularly interesting are the data on the reproductive toxicity of soil contaminants, which demonstrate the impairment of the reproductive capacity of both males and females; in addition, when the effects of pollutants on developing embryos were studied, serious morphological and molecular alterations were found. Overall, these damages demonstrate the high risk faced by these animals, which rely heavily on soil for reproduction. The toxic effects on the cells and tissues of adults, together with the impairment of the fecundity and development of the offspring, seriously endanger their survival, as well as that of all other vertebrates that share the habitat and type of reproduction with lizards. Several urgent actions can be taken to mitigate and prevent soil pollution and its impact on reptiles, such as adopting sustainable agricultural practices (organic farming, crop rotation, and integrated pest management), increasing recycling and reuse of wastepaper, plastic, glasses, and organic products, improving wastewater management, and, finally, using less hazardous chemicals.

The studies analyzed in this review confirm *Podarcis* lizards as a good model system in ecotoxicological and cytotoxicological research, providing accurate assessments of the general and/or specific effects of pollutants and clarifying the defense mechanisms activated in relation to different exposures. Finally, since the effects recorded in lizards have often been observed in other terrestrial vertebrates, it can be concluded that the results obtained from studies on these animals can also be translated to mammals.

## Figures and Tables

**Table 1:** List of soil pollutants whose effects have been studied in *Podarcis* lizards.

Type of Chemical	Chemical	Route of Exposure	References
Pesticides	Mixture	Natural occurring exposure	[32,33,34,35,36,37]
Pesticides	Mixture	Sprayed on subjects; contaminated food	[38]
Herbicides	Mixture	Natural occurring exposure	[39]
Herbicide	Diuron	Sprayed on water and soil	[40]
Herbicide	Glyphosate	Oral gavage	[41,42,43]
Fungicide	Methyl tiophanate	Sprayed on subjects; sprayed on water, soil, and food; intraperitoneal injection	[44,45,46,47,48]
Insecticide	Chlorpyrifos	Contaminated food	[49]
Insecticide	Imidacloprid	Oral gavage	[50]
Alkylphenols	Nonylphenol	Sprayed on food and water; intraperitoneal injection	[51,52,53]
Alkylphenols	Octylphenol and Nonylphenol	Intraperitoneal injection	[54,55,56]
Polychlorinated Biphenyls	Mixture	Contaminated soil or intraperitoneal injection	[57]
Metal	Cadmium	Contaminated food; contaminated drinking water; intraperitoneal injection	[29,58,59,60,61,62,63,64,65]
Metal	Cadmium	Incubation of in ovo embryos in contaminated soil	[66,67,68,69]

**Table 2:** Summary table of effects observed in *Podarcis* lizards following exposure to different groups of contaminants.

Contaminant Group	Effects Found
Pesticides	Alteration in androgen and estrogen receptors gene expression; Alteration of steroidogenesis; Alterations in adrenal and thyroid glands morphology and functions; Altered gene expression; Apoptosis; Cytotoxicity; Genotoxicity; Hepatocytes degeneration and vacuolation; Liver fibrosis; Oxidative stress; Reduced germ cells; Reduced reproductive output.
Endocrine Disrupting Chemicals	Alteration in androgen and estrogen receptors gene expression; Alteration of steroidogenesis; Alterations in adrenal and thyroid glands morphology and functions; Apoptosis; Cytotoxicity; Hepatocytes degeneration and vacuolation; Oxidative stress; Reduced germ cells; Reduced reproductive output; Tissue accumulation; Vitellogenin induction.
Metals	Altered gene expression; Alterations in brain morphology; Alterations in pituitary gland morphology and functions; Alterations of liver parenchyma; Alteration in hepatocytes morphology and functions; Apoptosis; Cytotoxicity; Developmental abnormalities; Egg absorption; Maternal transfer; Oxidative stress; Reduced reproductive output; Reduced hatching success; Tissue accumulation.

## Data Availability

No new data were created.
